# Altered hair root gene expression profiles highlight calcium signaling and lipid metabolism pathways to be associated with curly hair initiation and maintenance in Mangalitza pigs

**DOI:** 10.3389/fgene.2023.1184015

**Published:** 2023-06-07

**Authors:** Nadia Khaveh, Kathrin Schachler, Jan Berghöfer, Klaus Jung, Julia Metzger

**Affiliations:** ^1^ Research Group Veterinary Functional Genomics, Max Planck Institute for Molecular Genetics, Berlin, Germany; ^2^ Institute of Animal Breeding and Genetics, University of Veterinary Medicine Hannover, Hannover, Germany; ^3^Department of Biology, Chemistry and Pharmacy, Institute of Chemistry and Biochemistry, Freie Universität Berlin, Berlin, Germany

**Keywords:** calcium signaling, pig, Mangalitza, curly hair, transcriptome profiling, weighted coexpression network analysis (WGCNA)

## Abstract

Hair types have been under strong targeted selection in domestic animals for their impact on skin protection, thermoregulation and exterior morphology, and subsequent economic importance. In pigs, a very special hair phenotype was observed in Mangalitza, who expresses a thick coat of curly bristles and downy hair. Two breed-specific missense variants in *TRPM2* and *CYP4F3* were suggested to be associated with the Mangalitza pig’s hair shape due to their role in hair follicle morphogenesis reported for human and mice. However, the mechanism behind this expression of a curly hair type is still unclear and needs to be explored. In our study, hair shafts were measured and investigated for the curvature of the hair in Mangalitza and crossbreeds in comparison to straight-coated pigs. For molecular studies, hair roots underwent RNA sequencing for a differential gene expression analysis using DESeq2. The output matrix of normalized counts was then used to construct weighted gene co-expression networks. The resulting hair root gene expression profiles highlighted 454 genes to be significantly differentially expressed for initiation of curly hair phenotype in newborn Mangalitza piglets versus post-initiation in later development. Furthermore, 2,554 genes showed a significant differential gene expression in curly hair in comparison to straight hair. Neither *TRPM2* nor *CYP4F3* were identified as differentially expressed. Incidence of the genes in weighted co-expression networks associated with *TRPM2* and *CYP4F3,* and prominent interactions of subsequent proteins with lipids and calcium-related pathways suggested calcium signaling and/or lipid metabolism as essential players in the induction of the curly hair as well as an ionic calcium-dependency to be a prominent factor for the maintenance of this phenotype. Subsequently, our study highlights the complex interrelations and dependencies of mutant genes *TRPM2* and *CYP4F3* and associated gene expression patterns, allowing the initiation of curly hair type during the development of a piglet as well as the maintenance in adult individuals.

## 1 Introduction

The skin and its adnexa provide the first line of defense and protection for the inner part of the body, offering an interface for dynamic interactions with the environment as well as a unique characteristic of an individual’s phenotype (Suskind, 1977). In particular, hair roots have been under frequent investigation due to their important role in thermoregulation, skin protection and sexual dimorphism (Ling, 1970; Herrling et al., 2008; Liwanag et al., 2012; Nery et al., 2014; Collier and Gebremedhin, 2015). In domestic animals, the process of domestication and subsequent targeted breeding for traits meeting the production goals and housing conditions has resulted in a high variety of morphological attributes, including hair coat (Diamond, 2002; Meyer et al., 2002). This variation has demonstrated a high potential for providing suitable models for research in the context of skin diseases, syndrome-associated hair phenotypes or hair loss (Porter, 2003; Jiang et al., 2021; Lunney et al., 2021). Especially, the pig was shown to be a particularly suitable model for studying hair follicle morphogenesis and complex hair traits due to its unique similarity to human hair development (Mangelsdorf et al., 2014; Jiang et al., 2021). Different hair types have been observed to be exceptional in specific pig breeds, including curly hair as one of the most striking traits. Breeding records suggest only a few existing pig breeds with characteristically curly hair; the Turopolje pig, a nearly extinct breed from Croatia, the Mexican Cuino pig, the Brazilian Canastrão, as well as the endangered Mangalitza pig originated from the Carpathian basin (Rhoad, 1934; Lemus-Flores et al., 2015; Čandek-Potokar and Nieto, 2019). The Mangalitza was established by crossing four former breeds including the curly-haired Bakony and Alföldi, as well as the Szalonta and a small population of the Serbian Sumadia in the early nineteenth century (Egerszegi et al., 2003). This resulted in a very characteristic hair coat in the Mangalitza, which contains two types of hair, namely, bristles or “Borstenhaar” according to German terminology and downy hair (softer and thinner hair) or “Flaumhaar” (Constantinescu et al., 1942). Both hair types displayed a similar shape and subjected to seasonal hair growth and molting (Constantinescu et al., 1942; Egerszegi et al., 2003; Schachler et al., 2020).

A similar phenotype with characteristically curly hair has also been observed and investigated in several domestic animals such as horse (Blakeslee et al., 1943; Scott, 2004), dog (Cadieu et al., 2009; Salmela et al., 2019), cat (Filler et al., 2012; Gandolfi et al., 2013b; Manakhov et al., 2020), rabbit (Rafat et al., 2008), rat (Kuramoto et al., 2010), mouse (Campagna et al., 2008), cattle (Daetwyler et al., 2014; Braun et al., 2018), sheep (Kang et al., 2013), goat (Xiao et al., 2019), chicken (Ng et al., 2012; Dong et al., 2018; Chen et al., 2022), and Japanese quail (Tsudzuki, 2008). Furthermore, curly hair was found in human from different ethnicities (Thibaut et al., 2007; Shimomura et al., 2010; Xu and Chen, 2011).

In pigs, the curly hair phenotype was proposed to be a dominant trait in Mangalitza based on crossbreeding experiments, and associated with two missense variants SSC2*:g.61866070T>C* in *CYP4F3* and SSC13*:g.207222334G>C* in *TRPM2* (Schachler et al., 2020). It was suggested that the crossbreeding of different curly-haired populations for establishing the Mangalitza breed allowed an intermixture of the two different variants, resulting in a similar phenotype (Schachler et al., 2020). This heterogeneity in the development of curly hair was also observed in other species, including horses and cats (Gandolfi et al., 2010; Gandolfi et al., 2013a; Thomer et al., 2018; Manakhov et al., 2020). In particular, different types of keratin or keratin-associated proteins, as well as *LIPH* and *LPAR6* related to lipid signaling pathways, have been frequently associated with curly hair in domestic animals (Kuramoto et al., 2010; Diribarne et al., 2011; Gandolfi et al., 2013a; Gandolfi et al., 2013b; Daetwyler et al., 2014; Braun et al., 2018; Thomer et al., 2018). It was suggested that the curliness of the hair might be a result of an axial asymmetry in the differentiation program of the follicle, highlighted by different expression patterns of *KRT14* and *TGM1* on the concave side of the hair follicle (Bernard, 2003; Thibaut et al., 2005). So far, several studies investigated expression profiles of curly hair to explain the underlying mechanisms of intercellular signaling and pathways relevant to hair development and function (Kang et al., 2013; Gao et al., 2016; Thomer et al., 2018; Gao et al., 2021; Li et al., 2022). However, the gene expression patterns of curly hair in Mangalitza have not been explored so far.

In this study, we investigate gene expression profiles specific to Mangalitza curly hair in comparison to straight hair based on RNA sequencing (RNA-seq) data. In our analysis, we identified genes potentially responsible for shaping curly hair, the postnatal initiation of the phenotype, as well as the altered expression patterns due to seasonal changes. Furthermore, we explored the regulatory pathways for curly hair development and maintenance using weighted gene co-expression networks to identify the interaction of differentially expressed genes (DEGs) with the two variants in *TRPM2* and *CYP4F3* and further modules associated with the curly phenotype.

## 2 Methods and materials

### 2.1 Animals and samples for expression profiling

In this study, six curly-haired individuals (blond Mangalitza), and two straight-haired individuals (an Angeln Saddleback sow and a Minipig boar) were kept under the same environmental conditions throughout the year and monitored for their hair development (Supplementary Table S1). Mating one of these curly-haired Mangalitza with the straight-haired Angeln Saddleback sow resulted in a litter of eleven Mangalitza×Angeln Saddleback piglets. For RNA isolation, hair roots from bristles were sampled from all individuals from the shoulder area on the midsagittal plane on the same day in March under the same sampling conditions. Furthermore, five of the curly-haired Mangalitza as well as the straight-haired Angeln Saddleback sow were additionally sampled in June, August, September, and December. The Mangalitza×Angeln Saddleback piglets underwent sampling 20 days (in December) and 4 months after farrowing (in March). In addition, samples from five straight-haired individuals (one Angeln Saddleback, three Husum Red Pied and one Husum Red Pied×Angeln Saddleback crossbreed) were obtained from a different animal farm. Local temperatures at the time of all sampling sessions were recorded. All samples were collected in RNAlater (Qiagen, Hilden, Germany) and stored at −20°C. All animal experiments were performed according to the national and international guidelines approved by the animal ethics committee of the Lower Saxony state veterinary office Landesamt für Verbraucherschutz und Lebensmittelsicherheit, Oldenburg, Germany (registration number 33.9-42502-05-17A217). The reporting in the manuscript follows the recommendations in the ARRIVE guidelines (Percie du Sert et al., 2020).

### 2.2 Phenotype

**FIGURE 1:**
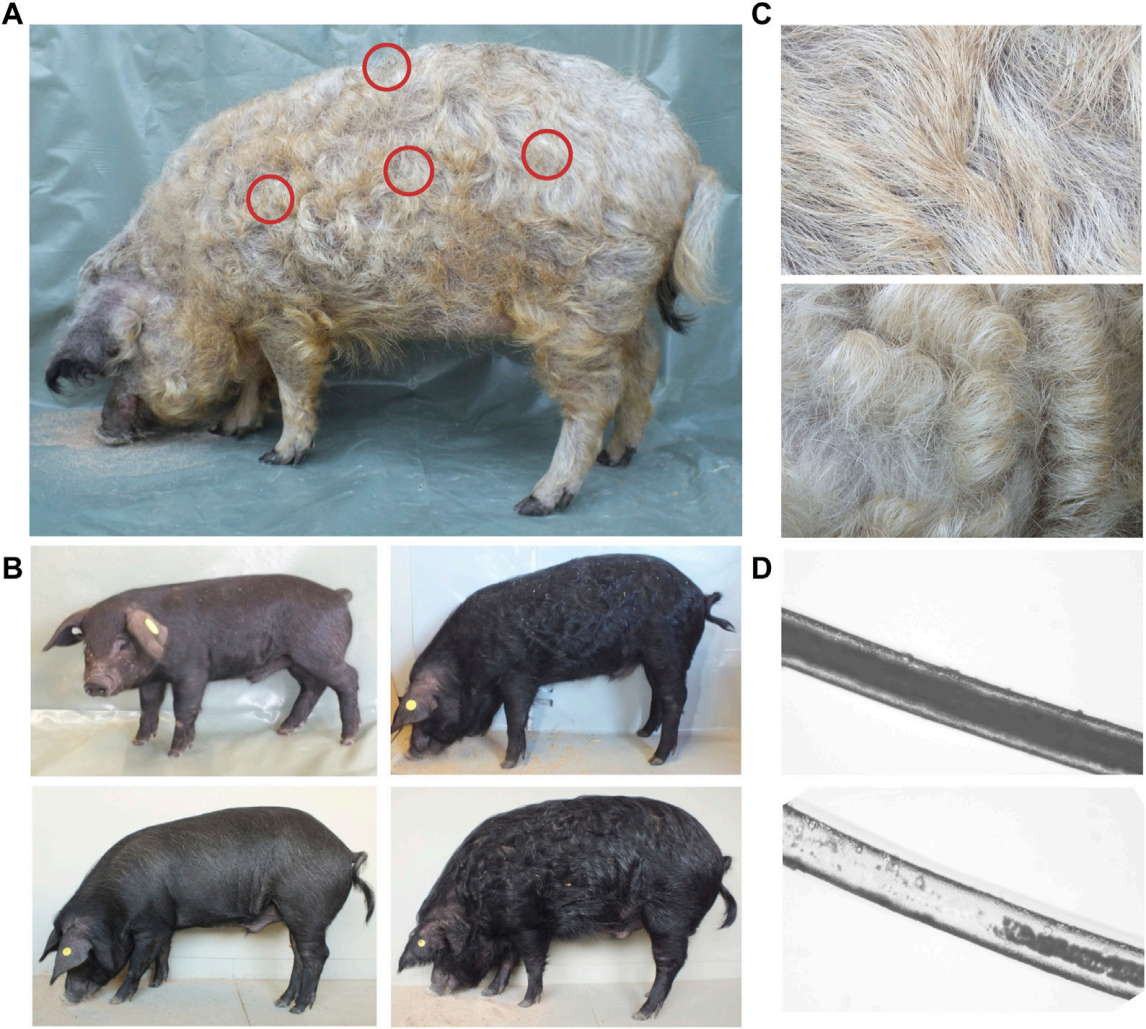
Overview of curly hair phenotype. **(A)** Sampling areas (shoulder, loin, buttock, and back) for determination of hair shape are designated. **(B)** Display of hair phenotype of a crossbreed pig at the age of 2 weeks in November (cold season, left, top), 4 months in March (cold season, right, top), 9 months in August (warm season, left, bottom), and 16 months in March (cold season, right, bottom). **(C)** Mangalitza hair in warm (top) and cold (bottom) seasons. In the cold season, a thicker coat with an increased curliness is observable. **(D)** The microscopic view (100x) of a bristle from Mangalitza in middle (top, medullated) and tip (bottom, fragmented medulla) position of the fiber.

In total, 15 bristles per area were obtained from shoulder (pectoral), loin (lateral abdominal), and buttocks (pelvic) at the articulatio humeri-level of the right side of the body as well as from the middle of the back (abdominal-dorsal) (Figure 1A) from all six curly-coated Mangalitza, 11 Mangalitza×Angeln Saddleback shoats, and two straight-coated individuals (Angeln Saddleback and Minipig). In addition, further 15 downy hair shafts were plucked from the loin from all six Mangalitza. On the day of collection, all hair shafts were treated and measured under the same environmental conditions according to the hair type-definition method suggested by De La Mettrie et al. (2007). Each hair shaft was fixed in-between two glass slides and investigated for the curvature of the hair, namely, the curve diameter (CD), using a template from Bailey & Schliebe (Bailey and Schliebe, 1986). Furthermore, the ratio of hair length when fully extended (*L*_*2*_) over its natural length (*L*_*1*_ *)* was determined as the curl index (*i* = *L*_2_ */L*_1_) (De la Mettrie et al., 2007). A larger curl index indicates curlier hair, whereas an index closer to one represents straighter hair. Moreover, as an additional parameter, the number of waves was detected by fixing the two ends of the hair with a tape and counting its ups and downs. The higher number of the “ups and downs”-count was defined as the number of waves.

Furthermore, all pigs in this study were genotyped for the two previously identified variants in *TRPM2* and *CYP4F3*, *SSC2:g.61866070T>C (CYP4F3) and SSC13:g.207222334G>C (TRPM2),* for curly hair in Mangalitza (Schachler et al., 2020).

### 2.3 RNA isolation and quality control

For RNA isolation, samples from bristles were washed once in ice-cold 1X PBS to remove any residual crystalized RNAlater. Hair roots were transferred into 2 mL tissue grinding CKMix50 tubes (Precellys, Bertin Corp, Rockville, United States) in the presence of 1 mL QIAzol lysis reagent (Qiagen) and placed into Precellys24 tissue homogenizer (Bertin Corp), programmed for one-time high energy of 5000 rpm for 10 s. After homogenization, the samples were incubated for 5 minutes at room temperature before being processed further with RNeasy Lipid Tissue Mini Kit (Qiagen). The integrity of the extracted bulk RNA was evaluated using RNA 6000 Nano kit (Agilent, Santa Clara, California, United States) on a 2,100 Bioanalyzer (Agilent). Only samples with RNA integrity number (RIN) > 7 were used for sequencing.

### 2.4 Sequencing and data preprocessing

RNA libraries, with equal RNA amounts, from five curly-coated Mangalitza, seven Mangalitza×Angeln Saddleback shoats, and seven straight-coated individuals (see Supplementary Table S1) were prepared using TruSeq Stranded Total RNA Library Prep Kit (Illumina, San Diego, United States) and indexed by NEBNext Multiplex Oligos for Illumina (Unique Dual Index Primer Pairs, NEB, Ipswich, United Kingdom). The prepared libraries were set in equimolar dilutions and submitted for sequencing for 66 million reads. In total, 32 libraries were sequenced on an Illumina NextSeq500 for 2 × 75 bp, further 15 libraries were sequenced on an Illumina NovaSeq6000 for 2 × 100 bp. In the next step, the binary base call (BCL) sequence output files were converted to FASTQ format and underwent quality control using FastQC version 0.11.8 (Andrews, 2010). FASTQ files were further processed using fastp version 0.20.0 (Chen et al., 2018) to control for adaptor content and discarding long stretches of N base >50 (*--n_base_limit 50*), 70% unqualified bases (unqualified_percent_limit 70), 1% complexity threshold (*complexity_threshold 1*) and low-quality reads (<phred score 5). Mapping was performed using STAR version 2.7.9a (Dobin et al., 2013) based on *Sus Scrofa*11.1 genome reference (Ensembl, database version 101.111). In addition, STAR *quantMode* was run to extract raw gene counts.

### 2.5 Differential gene expression analysis

Gene counts were analyzed based on a negative binomial distribution model provided by the R-package DESeq2 version 1.32.0 (Love et al., 2014). Preceding differential gene expression analysis (DEA), the quality was controlled for the transformed data based on *regularized logarithm (rlog)*, incorporating an estimated prior distribution of the sample differences (Love et al., 2014). Based on this, principal component analysis (PCA) was performed to control the samples spanned by their first two principal components and the overall effect of experimental covariates and batch effects.

DEAs were run to address two objectives: First, we hypothesized that a differential expression of exclusive gene clusters involved in shaping the hair can be found in curly-haired individuals when compared to straight-haired individuals (CvsSt). For this hypothesis, the phenotype and sequencer/read length of 19 individuals (7 straight-haired and 12 curly-haired) sampled in March were considered in the design formula (as potential batch effects) for DESeq modeling. Second, we proposed that the same gene clusters differentially expressed in curly-haired Mangalitza mentioned above might also be essential for the first-time initiation of a curly-hair in pigs, which are not purely Mangalitza but hybrids, and express the same curly-haired phenotype as well (SHvsP). For this analysis, seven Mangalitza×Angeln Saddleback crossbreeds at the age of 20 days (piglets) and 4 months (shoats) were compared by taking the age of the seven individuals as a variable factor in the dataset matrix of DESeq2.

The counts from both datasets under study were filtered to keep row sum >1 before they underwent DEA. The calculated raw *p*-values were adjusted according to Benjamini and Hochberg (Benjamini and Hochberg, 1995), and the false discovery rate (FDR) threshold was set to 0.05. Besides the significance threshold, selected genes were restricted based on a threshold of 2 for the absolute log2 fold change, i.e., |log2(FC)|>2.

In addition, a time-course analysis (TCA) was conducted to investigate differential gene expressions across months and subsequent temperature changes. Samples from five curly-haired and one straight-haired individuals from five different months (March, June, August, September, and December) were assigned to this approach. The samples of one of the curly-haired individuals obtained from June, September, and December did not reach an appropriate RIN and were excluded from the analysis. Therefore, the data of curly-haired individuals were compared to those from the straight-haired individual (Angeln Saddleback) by submitting them to a dataset matrix designed for phenotype and month with batch effect removal for sequencer/read length [R-packages DESeq2 and limma version, 3.48.3 (Love et al., 2014; Ritchie et al., 2015)]. Due to the lack of replicates in the straight-haired group, we analyzed the genes expressions (*sage.test)* from the statmod package for R [version 1.4.36, (Giner and Smyth, 2016)], for each time point, to compare the straight-haired sample with each curly-haired individual. Consequently, several *p-*values for each month were generated. The *p-*values were combined in parallel for each serial analysis into a single value using Simes combination methods.

### 2.6 Transcript identification and expression levels

Transcripts were identified using the cufflinks-ballgown pipeline. In the first step, the sorted bam files obtained from STAR were assembled into transcripts using cufflinks [version 2.2.1, (Trapnell et al., 2012)] and merged with the reference annotation into one gtf file to provide a reference annotation-based transcript assembly file. Next, a cufflinks-dependent tool, Table maker (version 2.1.1) was used to produce ctab files suitable as the input for the ballgown package for R [version 2.24.0 (Frazee et al., 2015)]. Gene name, transcript, and gene ID specific to cufflinks, chromosome, start, and end of transcription, number of exons and the length of the transcripts were exported and assigned to the significant DEGs identified by DESeq2 in the R environment.

### 2.7 Weighted co-expression network construction

Weighted correlation network analysis for co-expressions of genes was run using the WGCNA R package [(version 1.70–3) (Langfelder and Horvath, 2008)]. The output matrix of normalized counts from DESeq2 initial analysis, stabilized with *rlog*, was used to construct a gene co-expression network for both approaches CvsSt and SHvsP. Gene IDs with no read in any of the samples were removed from the dataset. To produce a scale-free topological overlapping matrix with minimum noise and data loss, the soft-thresholding power was calculated (*pickSoftThreshold* function). A soft-thresholding power value of six (*r* ^2^ = 0.90) was used for CvsSt analysis and four (*r* ^2^ = 0.88) for SHvsP. After the data was assigned to an adjacency matrix, a topological overlap matrix was configured, where the dissimilarity of each gene was correlated to all other genes. Next, the genes were clustered hierarchically (deepsplit = 2, automatic height cut>0.97) and placed in modules based on their similarities, which at a minimum contain 30 genes (*minModuleSize* = 30). The association of each gene with the trait (gene significance), correlation of module eigengene and the gene expression profile (module membership) were computed. Additionally, the correlation of each module with traits was tested using Student's *t*-test.

Moreover, we extracted the modules, which contained genes of two identified variants *TRPM2* and *CYP4F3*, *SSC2:g.61866070T>C (CYP4F3) and SSC13:g.207222334G>C (TRPM2)* (Schachler et al., 2020). Within each module, we filtered for the most central genes with the absolute value of gene significance measure >0.2 and the absolute value of module membership >0.8. The interaction of genes within these modules was visualized in a comprehensive network using cytoscape [version 3.9.1 (Shannon et al., 2003)].

In addition, the aforementioned genes affected by the Mangalitza-specific variants associated with curly hair shape, *CYP4F3* and *TRPM2*, were analyzed as the center of two separate networks, predicting the protein-protein interactions using STRING tool (version 11.5). These data were compared to the identified DEGs and the weighted correlation network identified by WGCNA.

### 2.8 Annotation and enrichment analysis

Functional annotation clustering and classification of the DEGs were done using the DAVID annotation tool (Huang et al., 2009). Furthermore, Ensembl IDs of DEGs were converted to gene names [*convert* function of gProfiler2 package (Kolberg et al., 2020)] in the R environment. In addition, gProfiler Orthology Search was used to find Ensembl gene identifiers for human orthologous genes. Based on human orthologues, EnrichR (Chen et al., 2013; Kuleshov et al., 2016; Xie et al., 2021) gene list enrichment analysis was applied using the gene-set libraries “GO Molecular Function 2021,” “GO Biological Process 2021” (Ashburner et al., 2000; Gene Ontology, 2021), “Human Phenotype Ontology” (Kohler et al., 2021), “KEGG 2021 Human” (Kanehisa and Goto, 2000; Kanehisa, 2019; Kanehisa et al., 2021), and “MGI Mammalian Phenotype Level 4 2021” (Smith and Eppig, 2009; Smith and Eppig, 2012; Smith and Eppig, 2015). The terms “hair” and “coat” along with other hair related terms, e.g., alopecia, hypotrichosis or hypertrichosis and vibrissae were specifically searched and filtered in all the generated annotation files.

### 2.9 Data validation

For validation of RNA-seq data, four curly- and three straight-haired individuals sampled in March were investigated. Two of these samples (one curly- and one straight-haired) already underwent differentially expressed genes analysis. Complementary DNA (cDNA) was synthesized using LunaScript RT SuperMix (NEB). An equal amount of cDNA (5 µg) from each sample was run on QuantStudio7 (Applied Biosystems, ThermoFisher, Waltham, United States) with set up for standard curve experiment in triplicates for TaqMan Gene Expression Assays (ThermoFisher). In total, five exemplary genes from the dataset, including *MMP9* (assay ID = Ss03392100_m1), *OLR1* (assay ID = Ss03392357_m1), *CD53* (assay ID = Ss06883185_m1), *TNFAIP6* (assay ID = Ss04246163_m1) and *LTF* (assay ID = Ss03384354_u1), along two housekeeping genes *GAPDH* (assay ID = Ss03375629_u1) and *ACTβ* (assay ID = Ss03376563_uH), as controls were tested. The amplification efficiency (90%-110%) and the correlation coefficient (*R* ^2^) for the standard curve of (>0.9) were evaluated. The expression levels were calculated by the ΔΔCT method (Livak and Schmittgen, 2001) using the R package “pcr” [version 1.2.2 (Ahmed and Kim, 2018)].

## 3 Results

### 3.1 Phenotype description

All investigated adult Mangalitza showed thick and long curly bristles as well as a thin downy hair (wool-like) covering their whole body (Figure 1A). In the Mangalitza×Angeln Saddleback crossbreeds, we observed the hair to be straight at the age of 20 days, but curly in the same individuals at 4 months’ age similar to Mangalitza (Figure 1B). Furthermore, both Mangalitza’s and crossbreeds’ hair revealed an increase in the degree of curliness in the cold season and a decrease in the warm season (Figure 1C). Under the microscope, the curly bristle shafts displayed a continuous medulla occupying more than 70% of the shaft’s width but a more fragmented medulla to no medulla at the tips (Figure 1D).

Measurements of individual hair fibers revealed an average curve diameter of 3.10 and 3.47 cm in the shoulder (pectoral) and loin (lateral abdominal) regions, and 4.07 and 4.45 cm in buttock (pelvic) and back (abdominal-dorsal) regions in curly-haired pigs. In comparison, the hair fibers from straight-haired pigs showed a significantly higher curve diameter (7.95–9.82 cm, *p-*value < 0.001) and were thus less curly in all investigated body regions (Supplementary Figures S1A–D). In addition, a significantly higher curl index, representing a higher degree of curliness of the hair fiber, was observed in curly-haired pigs (bristles) in all four sampled regions (*p-*value < 0.001, average index of 1.33–1.42, Supplementary Figures S1E–H). The number of waves ranged from 0 to 4 in curly-haired pigs, in contrast to 0-1 in hair fibers from straight-haired ones (Supplementary Figures S1I–L). Furthermore, the downy hair specifically found in Mangalitza showed an even more significant decrease in curve diameter (higher degree of curliness) and an increase in the number of waves compared to the curly bristles. However, no significant difference was detected in the curl index between downy hair and bristles (Supplementary Figure S2).

Further genotyping of all investigated pigs revealed all curly-haired Mangalitza and crossbreeds harboring to be heterozygous or homozygous mutant for the curly-hair-associated genotype C/C in SSC2:g.61866070T>C (*CYP4F3)* and heterozygous or homozygous wild type in SSC13:g.207222334G>C *(TRPM2,* see also Supplementary Table S1). All straight-haired individuals showed the homozygous wild type-genotype.

### 3.2 Genes differentially expressed in curly versus straight hair

**FIGURE 2:**
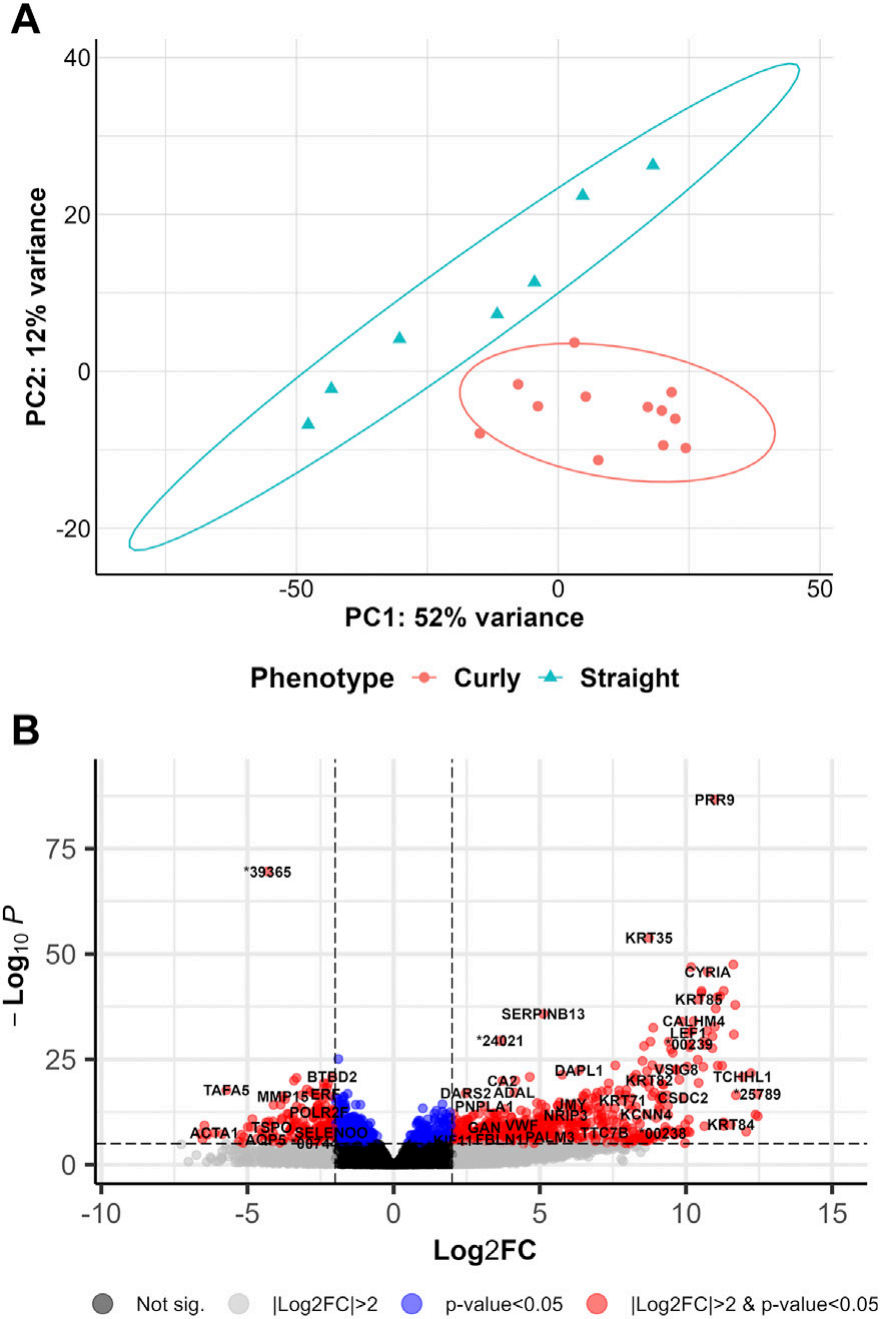
Overview of differentially gene expression analysis comparing curly hair pigs with straight hair pigs **(A)** PCA plot for curly-haired compared to straight-haired pig (CvsSt) datasets. **(B)** Volcano plot of the differentially expressed genes identified in CvsSt analysis.

DEA was run to address the potential gene regulatory mechanisms in shaping curly hair in pigs (curly versus straight (CvsSt), sampled in March). PCA revealed the first two principal components displaying a total of 64% variance across all 19 samples, with a distinct separation between the two phenotypes (Figure 2A). In total, 2,554 significant DEGs (padj <0.05 and |log2(FC)|>2) including 1,900 highly significant DEGs (padj <0.01 and |log2(FC)|>2) were identified (Figure 2B, Supplementary Table S2). In total, 2,036 of these DEGs were upregulated, whereas 518 DEGs were downregulated in curly-haired individuals. An intersection of these DEGs with transcripts identified with the cufflinks-ballgown pipeline revealed 19,602 and 4,293 transcripts assigned to the upregulated and downregulated DEGs, respectively (Supplementary Table S3). However, 229 as well as 81 transcripts of these groups were allocated to the unknown chromosome scaffolds.

Furthermore, a few DEGs were predicted to be interacting with one of the two genes harboring the candidate variants for curly hair in Mangalitza, namely, CYP4F3 and TRPM2 on protein level (STRING prediction); The prediction analysis indicated an interaction between the encoded proteins of nine DEGs (*CYP26B1, ALOX5, ALOX15, ALOX15B, ALDH1A1, MMP9, CYP2E1, HSD11B1* and *LTF*) with CYP4F3 (Supplementary Table S4). In addition, the encoded proteins of seven DEGs, *JAM3, TRPV2, SNAP25, ICAM2, CD53, MMP9 and ADAM8* were predicted to directly or indirectly interact with TRPM2. In addition, enrichment and clustering of the identified DEGs by DAVID functional annotation resulted in 63 groups, of which the second group with an enrichment score of 8.18 harbored keratin and keratin-related genes (Supplementary Table S5A). Further functional enrichment analysis of human orthologues of the DEGs highlighted biological processes including regulation of neurotransmitter or N-methyl-D-aspartate receptor activity (GO:0099601, GO:2000310), axonogenesis (GO:0007409) and axon guidance (GO:0007411, Figure 3, Supplementary Table S5B). The gene lists were enriched for terms such as woolly/waved hair (HP:0002224, MP:0000410), abnormal hair (follicle)/coat formation and morphology (e.g., HP:0011363, MP:0003809, MP:0010685, MP:0000377, MP:0003704), dry/fine hair (HP:0002213, HP:0011359), brittle hair (MP:0003848), rough coat (MP:0010179), curly/wavy vibrissae (MP:0001274, MP:0001279) and alopecia (MP:0000414).

**FIGURE 3:**
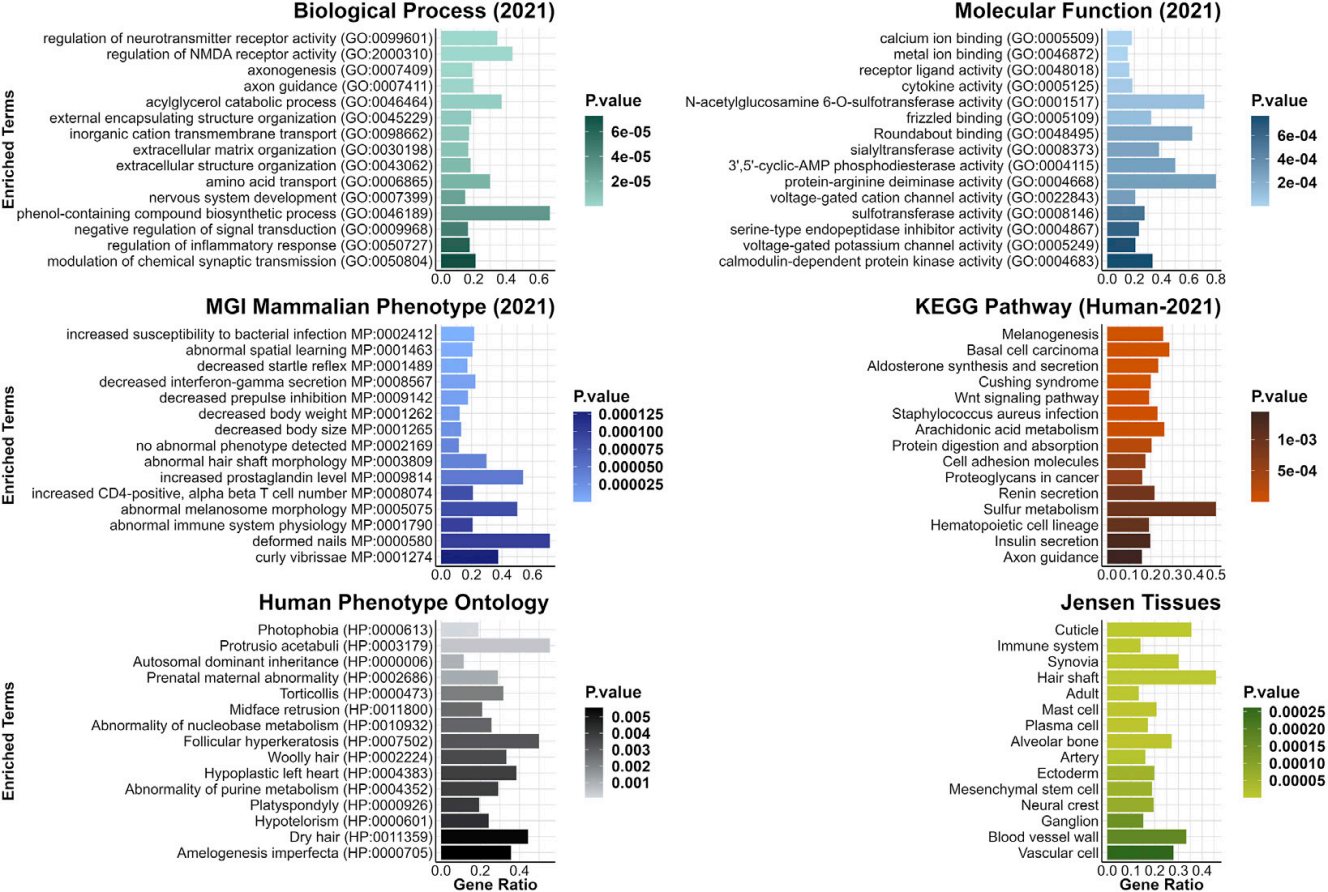
Top 15 enriched terms from gene list enrichment analysis using CvsSt data. Significantly differentially expressed genes identified in CvsSt analysis were run for gene list enrichment using the six gene-set libraries Molecular Function (2021), Biological Process (2021), KEGG Pathway (Human-2021), MGI Mammalian Phenotype (lvl4, 2021), Human Phenotype Ontology and Jensen Tissue.

**FIGURE 4:**
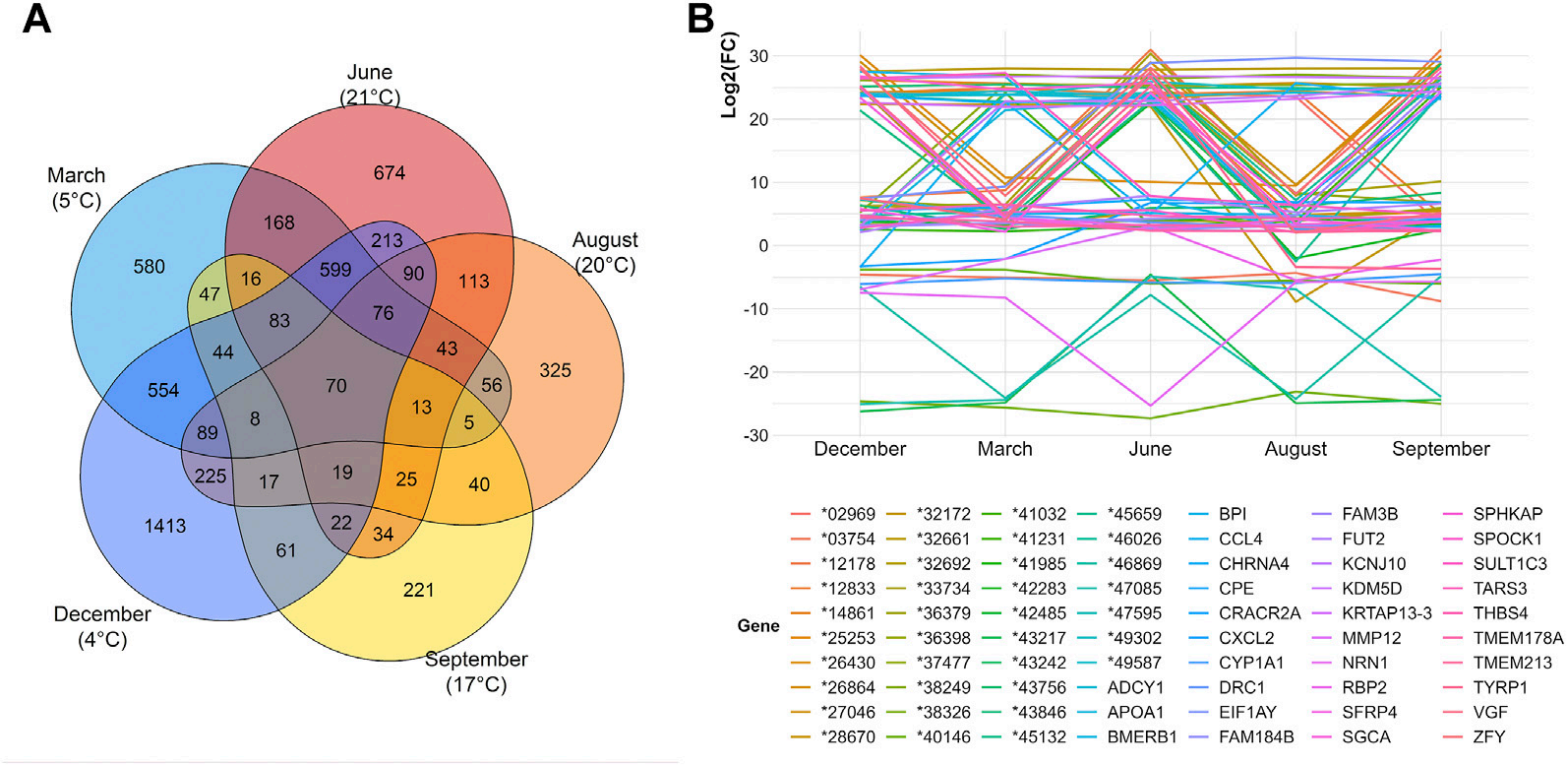
Overview of differential gene expression analyses in five different months. **(A)** Venn diagram of the number of differentially expressed genes in curly-haired compared to one straight-haired pig identified in five different months. The local temperature at the time of sampling is displayed. In total, 70 genes overlap within all tests. **(B)** Log2(FC) of the 70 genes with a differential expression across all 5 months displaying a variable pattern over the months.

As a next step, we performed TCA to investigate the differential gene expressions of curly versus straight hair across different months (March, June, August, September, and December) and subsequently different temperatures were investigated. For each month, different numbers of DEGs were identified, and the overlaps in between the timepoints revealed the DEGs shared among those months (Figure 4A). Overall, 70 DEGs were common among all investigated timepoints. These DEGs displayed prominent changes in gene expression over time with variable patterns of log2(FC) in different months (Figure 4B, Supplementary Table S6). In total, 27 of these genes were also found among the DEGs from CvsSt dataset, including *CRACR2A* (calcium release activated channel regulator 2A). Furthermore, neither *TRPM2* nor *CYP4F3* were differentially expressed in any of the investigated months.

### 3.3 Gene expression differences for curly hair initiation

**FIGURE 5:**
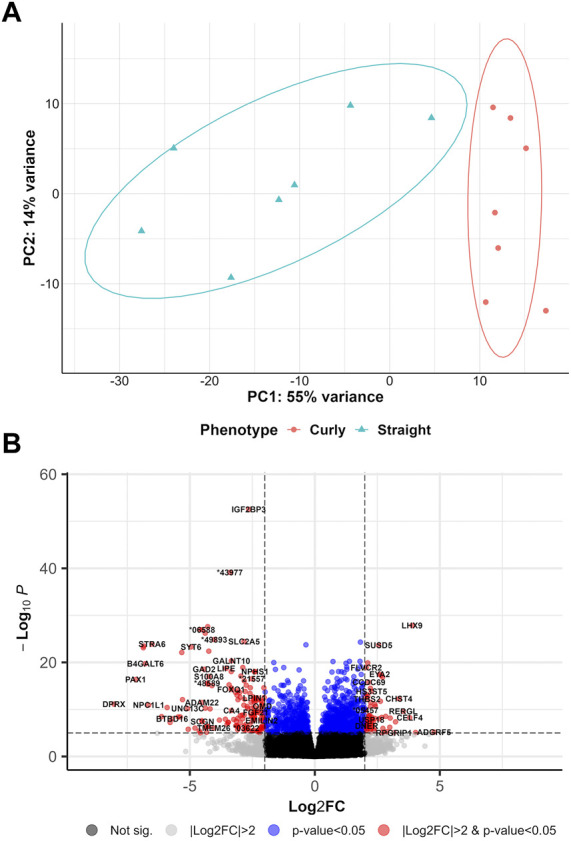
Overview of differential gene expression analyses comparing Mangalitza 1-month old piglet with four-month-old shoats. **(A)** PCA plot for curly-haired shoats compared to straight-haired piglet (SHvsP) datasets. **(B)** Volcano plot of the differentially expressed genes in SHvsP analysis.

To learn more about curly hair development, DEA was also performed to test for gene clusters, which might be essential for the first-time initiation of curly-hair weeks after birth and thus are also present in Mangalitza hybrid pigs expressing the same curly-hair phenotype as Mangalitza (shoat versus piglet, SHvsP). PCA based on the first two principal components showed a 69% variance between the newborn piglets and older shoats (Figure 5A). In total, 454 significant DEGs (padj<0.05 and |log2(FC)|>2) were identified, of which 194 genes were upregulated and 260 genes were downregulated (Figure 5B, Supplementary Table S7). Subsequently, 122 upregulated genes and 215 downregulated genes showed a highly significant differential gene expression with an adjusted *p-*value < 0.01. After intersecting the upregulated DEGs with the list of transcripts, we found a total of 1,471 transcripts assigned to 194 upregulated DEGs, as well as 1,653 transcripts for the downregulated DEGs (Supplementary Table S8).

Furthermore, intersecting the proteins of the identified DEGs with the protein interaction network centered at CYP4F3 revealed a direct relation with one protein encoded by the DEG *CYP4A90 (ENSSSCG00000003754)*. Moreover, the network centered on TRPM2 was found to harbor PLAUR, which was also identified among the DEGs.

**FIGURE 6:**
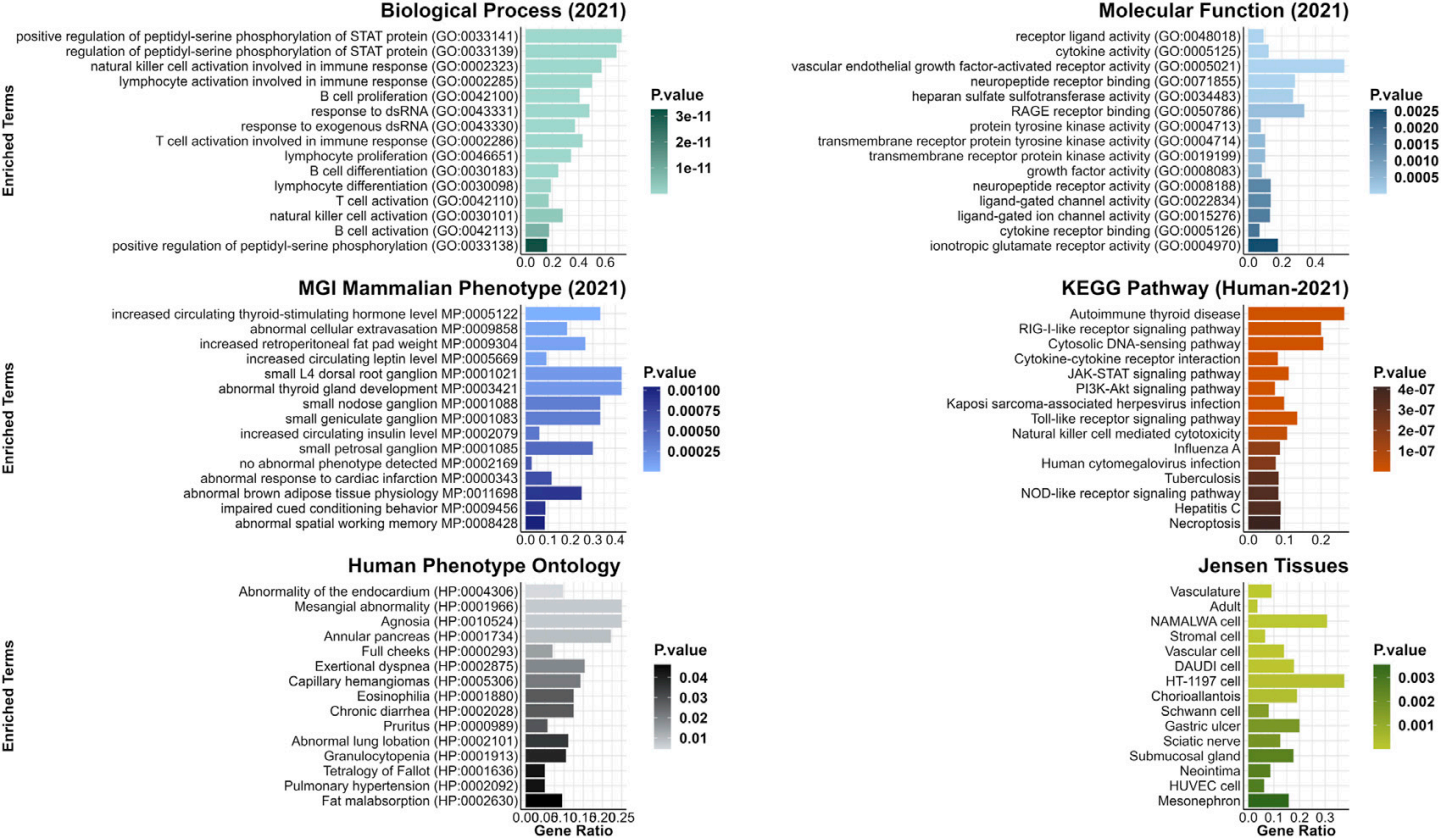
Top 15 enriched terms from gene list enrichment analysis using SHvsP data. Significantly differentially expressed genes identified in SHvsP analysis were run for gene list enrichment using the six gene-set libraries Molecular Function (2021), Biological Process (2021), KEGG Pathway (Human-2021), MGI Mammalian Phenotype (lvl4, 2021), Human Phenotype Ontology and Jensen Tissue.

In addition, enrichment and clustering of the identified DEGs by DAVID functional annotation resulted in eleven groups, including *ADGRs, THBS2* and *FBLN* in the first two groups (enrichment score 2.98 and 2.43) among others (Supplementary Table S9A). Human orthologues genes of the detected DEGs were found to be enriched for terms playing a role in biological processes such as positive regulation of peptidyl-serine phosphorylation of STAT protein (GO:0033141), regulation of peptidyl-serine phosphorylation of STAT protein (GO:0033139), natural killer cell activation involved in immune response (GO:0002323) and lymphocyte activation involved in immune response (GO:0002285). Furthermore, in phenotype gene-set libraries, genes were assigned to terms abnormal hair medulla (MP:0003812), and abnormal hair shaft morphology (MP:0003809) among others (Figure 6, Supplementary Table S9B).

**FIGURE 7:**
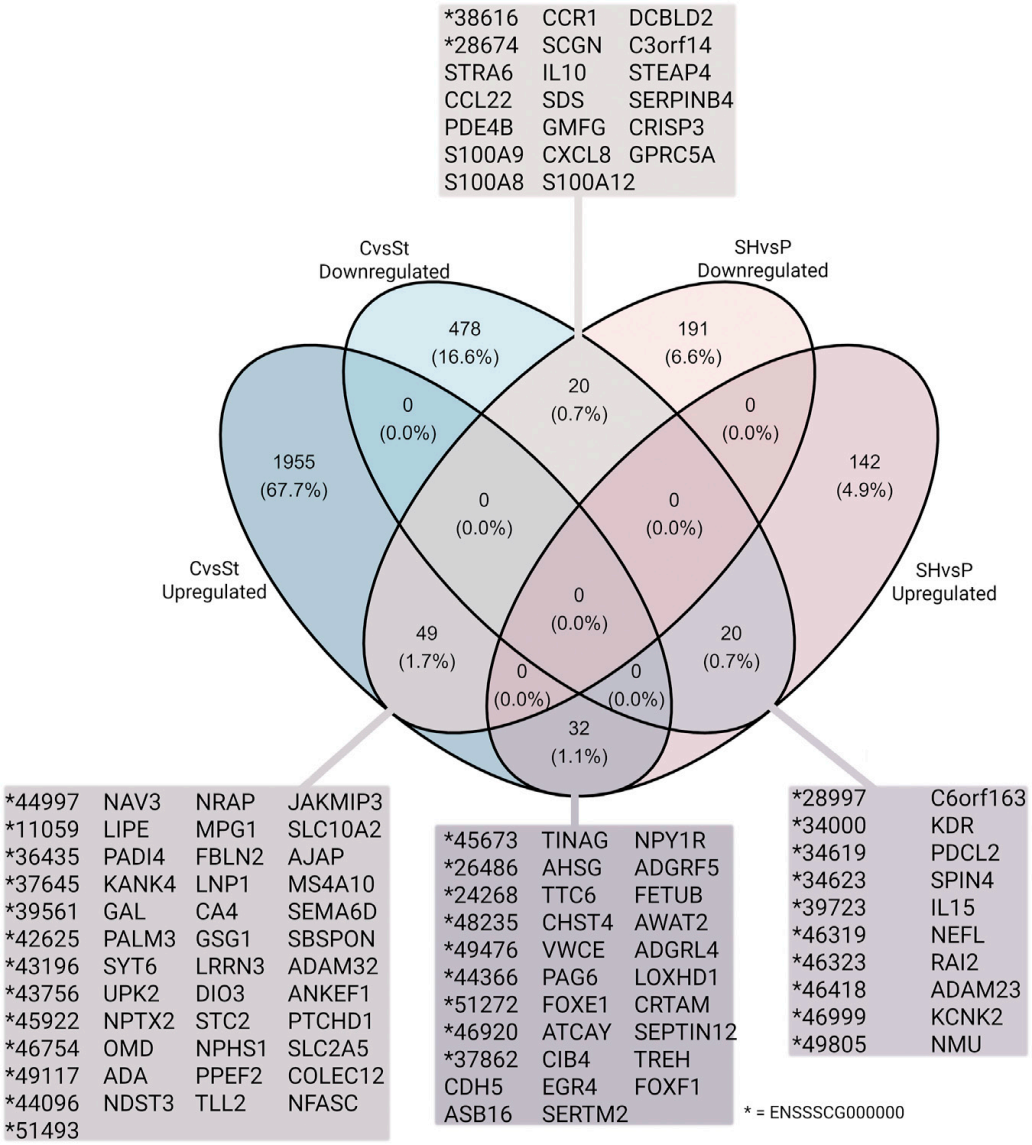
Logical relation between the two datasets of significantly differentially expressed genes. The number of up- and downregulated genes in CvsSt and SHvsP datasets, their overlap in percentage and subsequent gene names are displayed.

Finally, common DEGs were found in both datasets, CvsSt and SHvsP (Figure 7). In total, 49 downregulated and 32 upregulated DEGs (SHvsP) overlapped with the upregulated DEGs from the CvsSt dataset. Furthermore, 20 down- and 20 upregulated DEGs from the SHvsP dataset were also identified as downregulated DEGs in CvsSt.

### 3.4 Gene co-expression network analysis

**FIGURE 8:**
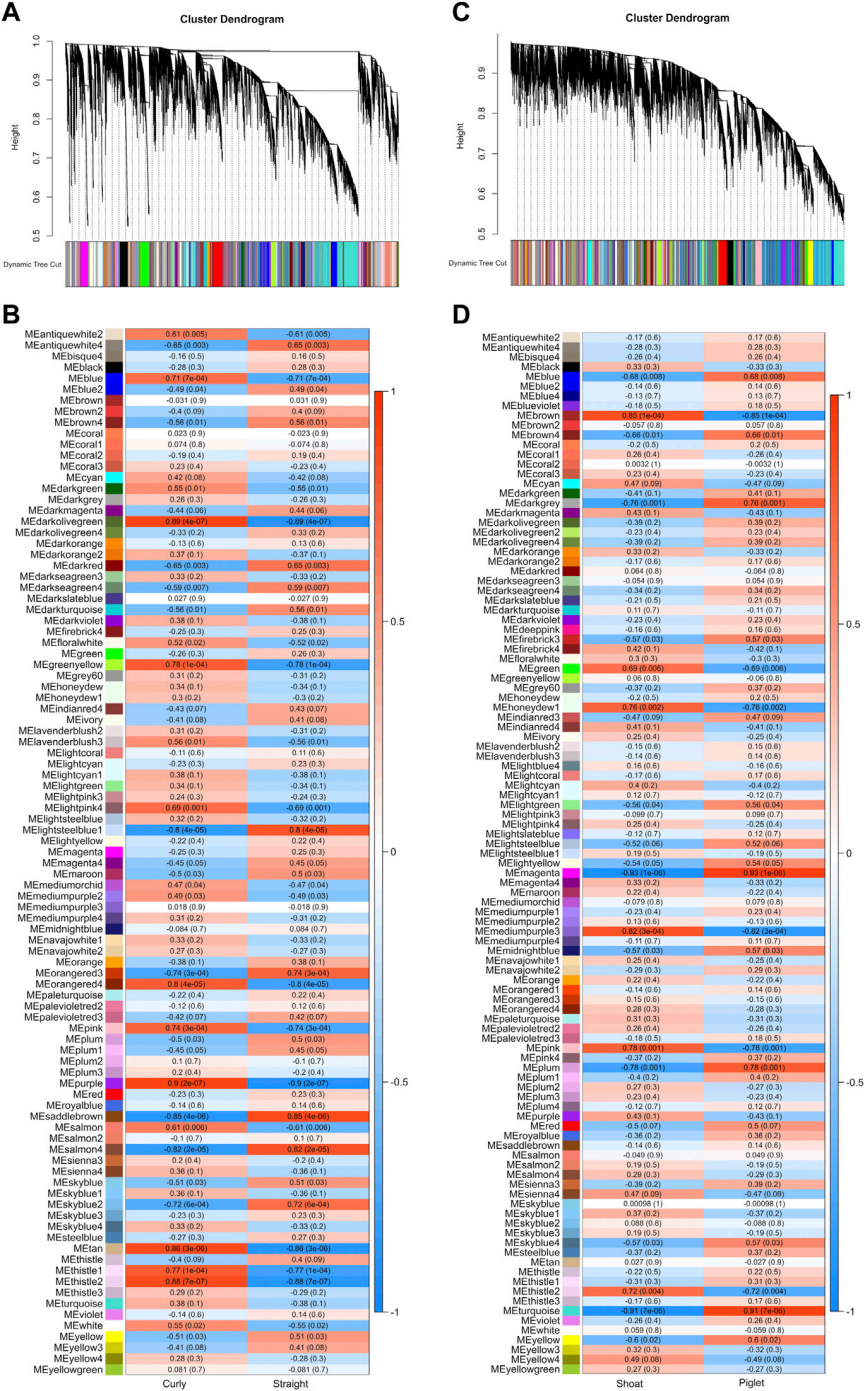
Weighted gene co-expression network analyses. **(A)** The hierarchical dendrogram shows branch cutting and module detection using the module eigengene in CvsSt and **(B)** the correlation of modules with curly and straight hair phenotype in CvsSt dataset. Similar **(C)** dendrogram as well as **(D)** correlation heat map for piglet and shoat phenotypes (SHvsP) were analyzed and displayed.

Weighted gene co-expression networks were constructed for a total of 24,200 expressed genes for 19 samples from the CvsSt dataset as well as 22,578 expressed genes for 14 samples from the SHvsP dataset. For CvsSt, hierarchical clustering revealed no outlier samples. In total, 95 co-expression modules were identified with all genes placed in a module (no gene was placed in module “grey”), which is reserved for unassigned genes (Figure 8A and Supplementary Table S10). The largest module contained 3,661 genes (turquoise), whereas the smallest module size was 31 (sienna4). In total, 28 modules had a strong correlation to curly phenotype with modules “purple” and “darkolivegreen” being at the top of this list, respectively (Figure 8B). Module “purple” showed a module-trait correlation = 0.9 (*p*-value = 2 × 10^-7^), containing 510 genes of which 157 were hub genes and 118 genes were identified as DEGs (Supplementary Table S11).

Next, we identified modules, which contained one of the two genes, *CYP4F3* and *TRPM2,* associated with curly hair in Mangalitza (Supplementary Table S13). In the CvsSt dataset, *CYP4F3* was assigned to module “sienna3,” which contained 179 genes (module-trait correlation = 0.2 and *p-*value = 0.4) and did not have any genes significantly correlated with the curly phenotype (no hub genes were identified within the assigned thresholds). None of the genes were found in the list of DEGs (CvsSt).

**FIGURE 9:**
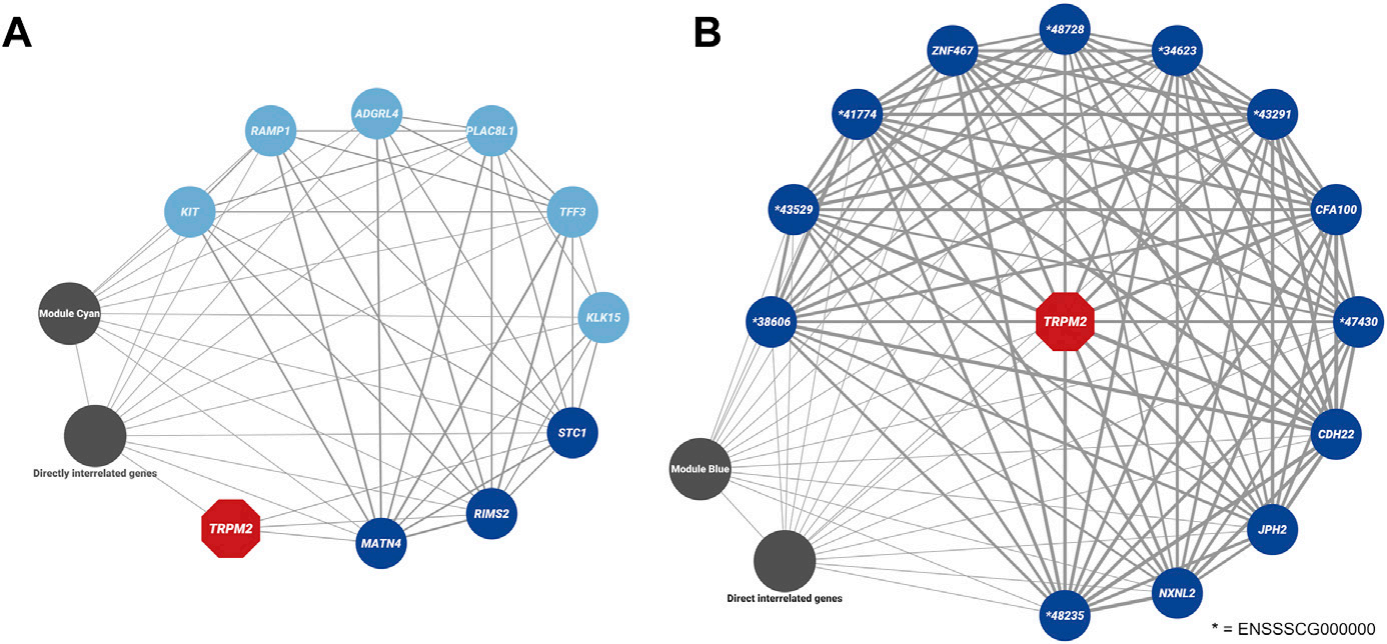
Weighted gene co-expression networks associated with *TRPM2* (highlighted in red). **(A)** *TRPM2* was identified in the module “cyan” in the dataset from CvsSt analysis (385 genes). Genes within the module, which were also found to be differentially expressed (light blue), differentially expressed and directly related to *TRPM2* (blue) or directly related to the differentially expressed genes (charcoal, “differentially interrelated genes”) are highlighted. All other genes of the module were collapsed. **(B)** In the dataset for SHvsP analysis, *TRPM2* was identified in the module “blue” (2,489 genes). Differentially expressed genes found within this module are displayed in blue. All these genes are predicted to be directly connected to *TRPM2*. All other genes are collapsed in this display (charcoal). The thickness of the lines presents the correlation between the two genes.

Furthermore, *TRPM2* was placed in the module “cyan” with 384 other genes of which 126 genes were significantly correlated to the curly hair trait (hub genes, Supplementary Table S11). In comparison to module “sienna3,” it had a stronger, but not significant correlation with the curly trait (module-trait correlation = 0.42 and *p-*value = 0.08). Among the genes within this module, nine genes were found to be differentially expressed (Figure 9A), of which two genes, *RIMS2* and *MATN4,* had an association with the curly phenotype (hub genes). Both *RIMS2* and *MATN4*, along with *STC1* showed a correlation coefficient above the threshold >0.02 with *TRPM2*.

The weighted co-expression network analysis for the SHvsP dataset clustered in a total of 107 modules with the smallest module (plum4) containing 32 genes and the largest module containing 4,199 genes (turquoise) (Figure 8C and Supplementary Table S10). In total, 19 modules showed a strong correlation to curly trait in shoats with module “magenta” (424 genes, module-trait correlation = −0.93 and *p-*value = 1e-06) and “turquoise” (4,199 genes, module-trait correlation = −0.91 and *p-*value = 7e-06) at the top of the list (Figure 8D). In module “magenta,” 12 hub genes and 21 DEGs were identified (Supplementary Table S12). Furthermore, in the SHvsP dataset, *TRPM2* was located in module “blue” with 2,489 genes, 644 of which were highly correlated to curly trait (hub genes). The module was negatively correlated to the curly trait in shoats (module-trait correlation = −0.68 and *p-*value = 0.008). Thirteen DEGs were found in this module (Figure 9B) of which two genes, namely, *CDH22* and *TFF3,* were hub genes. All 13 genes showed a correlation coefficient above the threshold >0.02 with *TRPM2*. In addition, we identified *CYP4F3* in module “turquoise,” the largest module as mentioned above, of which 1,271 genes were highly associated with the curly trait. In total, 284 genes of module “turquoise” were identified to be DEGs including 160 of these genes designated as hub genes.

Finally, *TRPM2*-and *CYP4F3*-associated modules were searched for genes found in both datasets as DEGs (CvsSt and SHvsP) (Table 1). We identified *ADGRL4* in the *TRPM2*-associated module “cyan” (CvsSt analysis), which was found to be upregulated in both CvsSt and SHvsP analysis as well as *ENSSSCG00000048235* in module “blue” (SHvsP analysis). In addition, we identified *ENSSSCG00000034623* in module “blue” to be upregulated in SHvsP and downregulated in CvsSt. Furthermore, 75 common DEGs were detected in the *CYP4F3*-associated module “turquoise” (SHvsP analysis) but none in module “sienna3” (CvsSt analysis).

**TABLE 1:** Common DEGs between the two datasets identified in WGCNA modules of interest. The overlap of *TRPM2*-or *CYP4F3*-associated modules from WGCNA with common DEGs from both datasets CvsSt and SHvsP are displayed.

Module (gene at the center)	Ensembl ID * = ENSSSCG000000 (gene name)
Cyan (*TRPM2)*	*03762 (ADGRL4)
Blue (*TRPM2)*	*48235, *34623
Sienna3 (*CYP4F3)*	*‾*
Turquoise (*CYP4F3)*	*01101 (SCGN), *24347 (CRISP3), *01909 (STRA6), *24633 (JAKMIP3), *02737 (CHST4), *26317 (SLC10A2), *02821 (CCL22), *26754 (ATCAY), *03018 (LIPE), *28204 (TREH), *03483 (PADI4), *03805 (PDE4B), *03821 (KANK4), *29249 (NAV3), *04663 (SEMA6D), *29284 (NPHS1), *06183 (SBSPON), *15807 (ADAM32), *16652 (LRRN3), *21255 (ADA), *39316 (GSG1), *11486 (C3orf14), *09115 (NDST3), *09649 (NEFL), *37987 (MS4A10), *11609 (FBLN2), *39651 (SLC2A5), *11799 (AHSG), *39673 (ASB16), *11800 (FETUB), *11964 (LNP1), *40397 (COLEC12), *11977 (DCBLD2), *40981 (GMFG), *12883 (GAL), *13767 (PALM3), *15100 (UPK2), *15137 (CRTAM), *15299 (STEAP4), *15652 (IL10), *23186 (CA4), *22951 (NFASC), *08073 (OMD), *36364 (EGR4), *08888 (NPY1R), *36865 (SDS), *08972 (PPEF2), *32433 (PTCHD1), *06590 (S100A8), *32857 (S100A12), *10507 (TLL2), *29592 (GPRC5A), *06758 (SYT6), *07933 (SEPTIN12), *35772 (CDH5), *37645, *11059, *39561, *28674, *28997, *46754, *43196, *46319, *38616, *45922, *42625, *43756, *44997, *39723, *33560, *49117, *37862, *49805, *51493

### 3.5 Data validation

Validation by real-time PCR of three randomly selected genes designated as DEGs in CvsSt analysis (*MMP9, CD53, LTF*) as well as further two genes not identified as DEGs *(OLR1* and *TNFAIP6)* confirmed the expression pattern identified in our DEA based on RNA-seq data (Supplementary Table S13). Significant downregulation of *MMP9*, *CD53*, *LTF* and *OLR1* (*p*-value < 0.05) in curly-haired individuals compared to the straight-haired individuals in March was observed. Although *OLR1* did not reach significance in our DEA (padj = 0.08), it was identified to be downregulated 3.6-fold with a *p-*value < 0.05. Furthermore, we confirmed no differentiation of *TNFAIP6* gene expression.

## 4 Discussion

In the present study, we characterized the distinctive curly hair type in Mangalitza and gained new insights into the underlying complex interplay of differentially expressed genes and the two Mangalitza-specific missense variants in *TRPM2* and *CYP4F3*. The degree of curliness was observed to be even higher in downy hair in Mangalitza, a unique hair type exclusively observed in this breed and F_1_ generations of Mangalitza-crossbreeds (Schachler et al., 2020). Furthermore, we highlighted the eligibility of the descriptive parameters, curve diameter, curly index, and the number of waves, for estimating the degree of curliness in pig hair. These parameters were originally developed for phenotyping human hair fibers (Bailey and Schliebe, 1986; De la Mettrie et al., 2007) and were previously used to categorize hair fibers from different parts of the body in pigs (Jiang et al., 2021).

In the curly hair roots, we found a wide range of differential gene expression patterns in comparison to straight hair. Our enrichment analysis suggested that these DEGs predominantly play a role in hair morphogenesis, homeostasis, development and shaping, with particular evidence of the involvement of WNT and hippo signaling pathway-associated genes such as *WNT3, WNT8B, WNT10B, WNT11, TCF7, DVL2, BAMBI, LEF1, FZD10, NKD2* and *APC*, as well as genes involved in arachidonic acid metabolism (for example, *ALOX15*, *ALOX5*, *CYP2E1*, *GGT5* and *GPX3),* cell adhesion or cytokine activities (Jindo et al., 1994; Kaplan and Holbrook, 1994; Gat et al., 1998; Huelsken et al., 2001; Andl et al., 2002; Zhang et al., 2009; Munkhbayar et al., 2016; Lee et al., 2021; Li et al., 2022). Furthermore, the differential expression of *EDAR* and *SHH* suggested all three pathways, Wnt, NF-κB/Edar, and SHH, to be associated with curly hair development in pigs, as they were reported to be the most crucial signaling pathways for hair cycling, hair follicle development, and for driving progenitor proliferation (Callahan and Oro, 2001; Van Mater et al., 2003; Botchkarev and Fessing, 2005). Subsequently, we assume that these factors might also be relevant for the temperature-dependent seasonal hair growth associated with a significant increase in the curliness of the hair in colder seasons in Mangalitza and Mangalitza-crossbreeds (Watson and Moore, 1990; Egerszegi et al., 2003; Schachler et al., 2020). Our data from TCA revealed gene expression profiles under constant changes during the year. One of the 27 overlapping genes in-between the two analyses was *CRACR2A*, *the calcium release activated channel regulator 2A*. However, due to the limited number of controls in this specific approach, the individual variations in gene expressions have to be interpreted with care as they followed the annual cyclic intervals only to some extent, which might be a result of an intermingled pattern with the additional individual- or breed-specific effects. Nevertheless, these findings suggest that curly hair development is governed by season-dependent effects and complex gene expression patterns. In various studies in domestic animals, many different variants were identified to be causative for curly hair, enabling the maintenance of this phenotype even in each hair growth cycle (Cadieu et al., 2009; Gandolfi et al., 2010; Kuramoto et al., 2010; Gandolfi et al., 2013a; Gandolfi et al., 2013b; Morgenthaler et al., 2017; Braun et al., 2018; Thomer et al., 2018; Salmela et al., 2019; Manakhov et al., 2020). This was also suggested for the two missense variants in *CYP4F3* and *TRMP2,* which were found to be Mangalitza-specific and due to the gene’s function know in other species potentially associated with curly hair shape in pigs (Schachler et al., 2020). These variants were confirmed in our investigated Mangalitza and Mangalitza-crossbreeds. However, in accordance with previous investigations (Schachler et al., 2020), we found the hair fibers in the Mangalitza-crossbreeds to be still straight at 20 days, but curly at 4 months of age. As both *CYP4F3* and *TRMP2* were not differentially expressed in our analysis of the two age groups, we suggest this might be a result of a delay in a downstream activation, up- or downregulation by certain pathways, such as cytokine-cytokine receptor, JAK-STAT or PI3K/Akt signaling pathway, which we found highly enriched in our gene sets. It was postulated that these pathways play an essential role in the onset of anagen, keratinocyte growth, and differentiation as well as hair follicle regeneration by either positive or negative feedback mechanisms (Bernard, 2003; Calautti et al., 2005; Harel et al., 2015; Chen et al., 2020). In particular, the differentiation of keratinocytes, their initiation of rapid mitosis and/or keratinization were suspected to be important to determine the curly hair fiber shape (Thibaut et al., 2005; Thibaut et al., 2010; Sriwiriyanont et al., 2011). Several studies proposed that the production of keratins by keratinocytes and their expression in an irregular pattern in hair cortex (KRT38), cuticle (KRT82), outer or inner root sheath (KRT14, KRT74, KRT71) results in an asymmetrical development of the hair fibers and subsequently a curly phenotype (Thibaut et al., 2005; Thibaut et al., 2007; Shimomura et al., 2010; Thibaut et al., 2010; Fujimoto et al., 2012). Furthermore, it is noteworthy to mention that keratinocyte proliferation and differentiation are balanced by calcium signaling (Hennings et al., 1980; Li and Dong, 2016), a pathway, which we found to be enriched in our analysis. Thus, we assume that such a patterning process in the hair might be activated by *TRPM2*, a non-selective cation channel with high affinity to ionic calcium (Li and Jiao, 2020). It is interesting to note that in shoats who expressed curly hair for the very first time, we identified an upregulation of *KRT4,* which is known to be involved in the construction of hair fibers (Zhou et al., 2018) and thus might play a role in the initiation of curly hair. In addition, our co-expression networks associated with *TRPM2* gave further evidence for the involvement of genes with dependency on ionic calcium, as we found *JPH2*, a Ca^2+^ channel regulator (Landstrom et al., 2007), and *CDH22*, a calcium-dependent cell adhesion molecule (Cartwright et al., 2010), among 13 genes, which were also differentially expressed between the two age groups. Furthermore, our findings of a strong association of co-expression networks of *CYP4F3* with curly phenotype in shoats suggested various of the 281 DEGs promote direct interactions with lipids, such as *FOXQ1* and *SOAT1* as regulatory elements in hair shaft medulla formation and its internal lipid metabolism (Hong et al., 2001; Wu et al., 2010; Wu et al., 2013; Potter et al., 2015). Further genes in these data sets were identified as coding for glycosaminoglycan enzymes, which were shown to be essential parts of the extracellular matrix and were proposed to affect the diffusional mobility and subsequent dynamics of lipids by binding to these molecules in membranes (Raman et al., 2005; Sahoo and Schwille, 2013). Studies in hair shafts highlighted their role in hair follicle proliferation and protection as early as in the prenatal stage (Malgouries et al., 2008; Gao et al., 2016), and suggested the products of glycosaminoglycan enzymes to support the adhesion of hair cortex to the cuticle (Takahashi and Yoshida, 2014). Thus, given the catalytic activity of CYP4F3 for the oxidation of arachidonic acid, an essential fatty acid (Jarrar and Lee, 2019), we assume that the curly hair initiation might be supported by gene products involved in lipid metabolism. The targeted selection of the Mangalitza for its two major characteristics, namely, the curly hair and the fatty type (Switonski et al., 2010), potentially resulted in a linked inheritance and dependency of both traits. However, we cannot exclude the possibility that this gene enrichment for lipid metabolism is due to the high selection for fat deposition in Mangalitza only and is therefore masking gene effects related to curly hair development.

Furthermore, in pigs with fully developed curly hair, we expected a wide range of co-expressed genes to play a role in the maintenance of the curly hair shape in an interconnected system with mutant *TRPM2* and *CYP4F3*. The four genes *MATN4, KIT, RAMP1*, and *KLK15* among nine genes, which we identified as differentially expressed in CvsSt analysis and co-expressed with *TRPM2,* were reported to be prevalent in hair follicles and suggested to play a role in the regulation of cellular growth (Mates et al., 2004; Komatsu et al., 2005; Martinez-Martinez et al., 2012; Sun et al., 2020). In addition, as discussed above for the initiation of curly hair, we found further involvement in calcium-dependent regulation of some of these DEGs including *STC1*, which was shown to play a role in the quiescence-proliferation balance of Ca^2+^ transporting epithelial cells (Li et al., 2021), *RAMP1*, which was found to target calcium-sensing receptors to transfer them from the endoplasmic reticulum towards the Golgi complex (Bouschet et al., 2005; Martinez-Martinez et al., 2012) and *ADGRL4*, harboring a calcium-binding EGF domain (Sheldon et al., 2021). Subsequently, we assume that a functional relationship of these factors with the cation channel encoded by *TRPM2* (Li and Jiao, 2020) might explain the co-expression of these genes and their joint association with the curly hair trait. Furthermore, *ADGRL4* represents a promising candidate, which might be associated with the temperature-dependent shaping of the curly hair as it was suggested to play a role in angiogenesis in the endothelial cells (Masiero et al., 2013; Favara et al., 2021), a process which is upregulated in response to radiation and heat (Kim et al., 2006). As this gene was also differentially expressed in shoats, even though not co-expressed with *TRPM2,* it might be involved in both curly hair initiation and maintenance. In addition to that, we found CYP4F3 to be predicted to interact with proteins encoded by nine of our identified DEGs, mostly involved in lipid metabolism, but did not find any DEGs in the *CYP4F3-*associated co-expression networks. This leads us to the suggestion that further co-expression networks independent of *CYP4F3* or *TRMP2*, particularly those which were highly associated with the investigated trait, might play an additional role in the maintenance of curly hair in Mangalitza.

In conclusion, the present study suggests curly hair initiation and maintenance in Mangalitza to underlie complex gene expression patterns prominently linked to the two candidate genes *TRPM2* and *CYP4F3*. Our data provide evidence for a delayed first-time appearance of curly hair in crossbreed piglets due to downstream effects on hair-developmental signaling pathways. *TRPM2*-and *CYP4F3-*associated co-expression networks were found to be quite prominently interconnected with DEGs in this initiation phase, suggesting the curly hair patterning process to be activated and further implemented in a calcium signaling- and/or lipid metabolism-dependent manner. This dependency on ionic calcium was also proposed for several genes, which we identified as DEGs for the maintenance of curly hair and were among the co-expression networks associated with *TRPM2.* We particularly highlight *ADGRL4* as a potentially relevant player in both initiation and maintenance of curly hair in association with temperature-dependent changes in hair shape across different seasons. Our data suggest various genes to be involved in the development of curly hair phenotype and emphasize the importance of further exploring the role of *CYP4F3*- and *TRMP2*-dependent or -independent effects in the future.

## Data Availability

The datasets presented in this study can be found in online repositories. The names of the repository/repositories and accession number(s) can be found below: https://www.ebi.ac.uk/ena, PRJNA862648.
