# Supplementary figures and images for: Altered hair root gene expression profiles highlight calcium signaling and lipid metabolism pathways to be associated with curly hair initiation and maintenance in Mangalitza pigs
Source: Front Genet. 2023 Jun 7;14:1184015. doi: 10.3389/fgene.2023.1184015 (PMC10282778; doi:10.3389/fgene.2023.1184015)

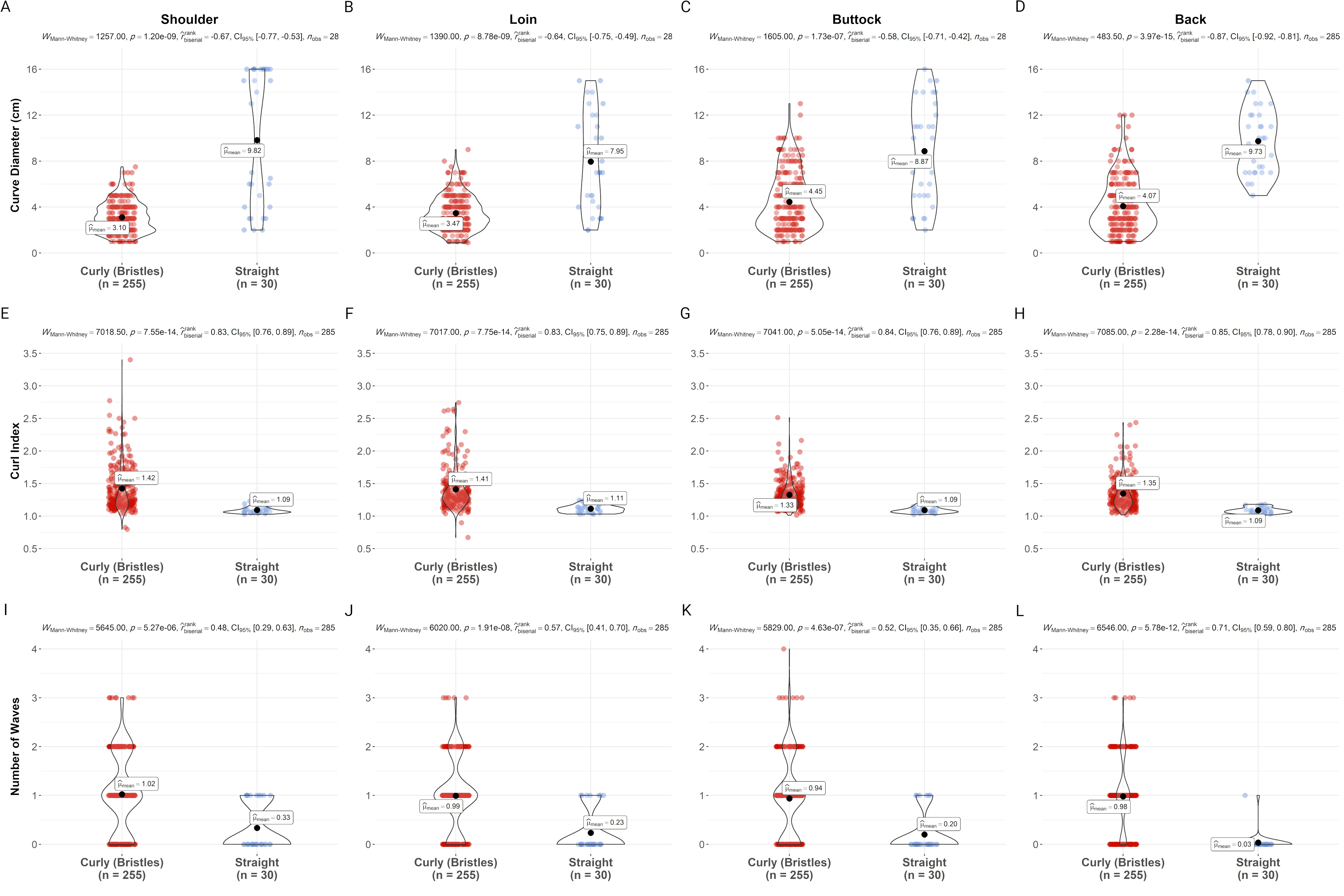

Supplement: Supplementary file 4 [file Image1.JPEG]

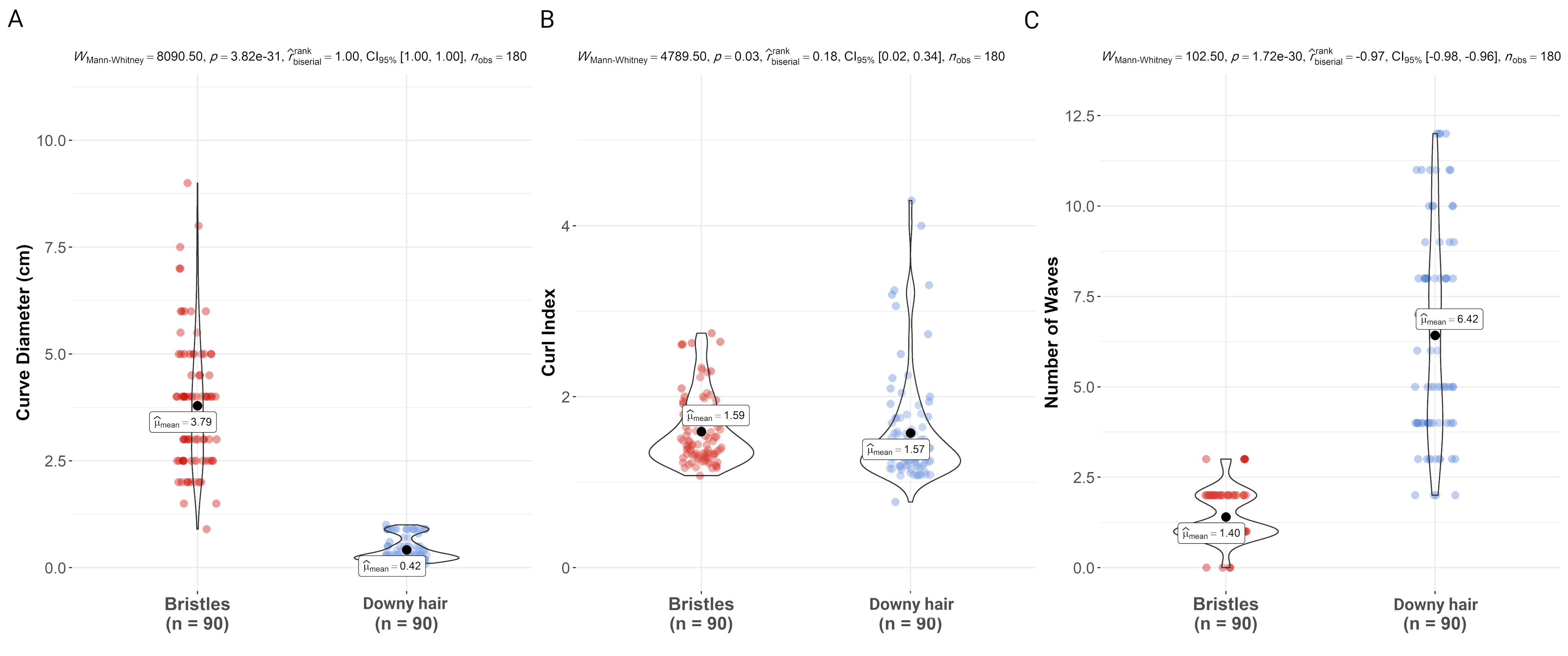

Supplement: Supplementary file 5 [file Image2.JPEG]
