# Supplementary material for: Altered hair root gene expression profiles highlight calcium signaling and lipid metabolism pathways to be associated with curly hair initiation and maintenance in Mangalitza pigs
Source: Front Genet. 2023 Jun 7;14:1184015. doi: 10.3389/fgene.2023.1184015 (PMC10282778; doi:10.3389/fgene.2023.1184015)
Supplement: Supplementary file 9 [file Table13.DOCX]

**Table S13 Validation of differential expression analysis (DEA) results.** A comparison between DEA (log2(FC)) and real-time PCR (relative expression) results for five genes is displayed. Those genes identified as DEGs in the study are highlighted bold. lfcSE = standard error of the log2(FC) estimate. stat = Wald statistic. CI = confidence interval.

|  | Gene Name | *LTF* | | *TNFAIP6* | | *CD53* | | *OLR1* | | *MMP9* | |
| --- | --- | --- | --- | --- | --- | --- | --- | --- | --- | --- | --- |
| DEA | **BaseMean** | 124.6863 | | 1.227093 | | 17.24967 | | 1.565943 | | 20.80928 | |
|  | **Log2(FC)** | -4.20488 | | -2.44293 | | -4.07522 | | -3.58242 | | -3.10577 | |
|  | **lfcSE** | 0.863672 | | 1.566098 | | 1.185344 | | 1.719718 | | 1.05.497 | |
|  | **Stat** | -4.8686 | | -1.55988 | | -3.438 | | -2.08314 | | -2.94247 | |
|  | **p-value** | **1.12×10^-6^** | | 0.118787 | | **0.000586** | | 0.037238 | | **0.003256** | |
|  | **padj** | **2.05×10^-5^** | | 0.215066 | | **0.003516** | | 0.088349 | | **0.013613** | |
| Real Time qPCR | **Reference Gene** | *GAPDH* | *ACTB* | *GAPDH* | *ACTB* | *GAPDH* | *ACTB* | *GAPDH* | *ACTB* | *GAPDH* | *ACTB* |
|  | **Calibrated** | 2.561818 | 3.401648 | -1.02272 | -0.50026 | 1.074778 | 1.638101 | 1.234854 | 1.808965 | 2.026916 | 2.601145 |
|  | **Relative expression** | 0.169362 | 0.094624 | 2.031746 | 1.414472 | 0.474744 | 0.321279 | 0.424885 | 0.285396 | 0.245379 | 0.164808 |
|  | **Error** | 1.642218 | 1.671736 | 1.624809 | 1.643696 | 0.97724 | 1.093208 | 1.46235 | 1.521409 | 1.105003 | 1.078828 |
|  | **Lower 95% CI** | 0.054257 | 0.0297 | 0.658799 | 0.452681 | 0.241147 | 0.150589 | 0.154192 | 0.099416 | 0.114077 | 0.078022 |
|  | **Upper 95% CI** | 0.528656 | 0.30147 | 6.265934 | 4.419735 | 0.934626 | 0.685442 | 1.1708 | 0.819289 | 0.527809 | 0.348126 |
|  | **p-value** | **0.000186** | **1.81×10^-5** | 0.159252 | 0.522002 | **0.031831** | **0.004845** | **0.020755** | **0.002963** | **0.005884** | **0.000814** |
