# Supplementary material for: Altered hair root gene expression profiles highlight calcium signaling and lipid metabolism pathways to be associated with curly hair initiation and maintenance in Mangalitza pigs
Source: Front Genet. 2023 Jun 7;14:1184015. doi: 10.3389/fgene.2023.1184015 (PMC10282778; doi:10.3389/fgene.2023.1184015)
Supplement: Supplementary file 15 [file Table10.DOCX]

**Table S****10** **Number of genes in weighted co-expressed network modules in CvsSt and SHvsP datasets**.

| **Module color** | **Number of genes** | **Module color** | **Number of genes** | **Module color** | **Number of genes** |
| --- | --- | --- | --- | --- | --- |
| **Analysis: CvsSt** |  |  |  |  |  |
| turquoise | 3661 | sienna3 | 179 | orangered3 | 86 |
| blue | 2215 | yellowgreen | 177 | plum | 86 |
| brown | 1302 | skyblue3 | 176 | lightcoral | 80 |
| yellow | 818 | orangered4 | 172 | indianred4 | 79 |
| green | 709 | plum1 | 172 | firebrick4 | 77 |
| red | 701 | mediumpurple3 | 165 | darkolivegreen4 | 76 |
| black | 594 | lightsteelblue1 | 160 | brown2 | 71 |
| pink | 588 | lightcyan1 | 156 | blue2 | 66 |
| magenta | 527 | ivory | 154 | darkviolet | 65 |
| purple | 510 | floralwhite | 153 | plum3 | 63 |
| greenyellow | 492 | darkorange2 | 150 | thistle3 | 62 |
| tan | 455 | brown4 | 140 | salmon2 | 58 |
| salmon | 436 | bisque4 | 133 | thistle | 58 |
| cyan | 385 | darkslateblue | 130 | palevioletred2 | 51 |
| midnightblue | 372 | plum2 | 130 | lightpink3 | 50 |
| lightcyan | 357 | thistle2 | 128 | magenta4 | 50 |
| grey60 | 297 | thistle1 | 124 | navajowhite1 | 50 |
| lightgreen | 294 | salmon4 | 120 | lavenderblush2 | 47 |
| lightyellow | 274 | palevioletred3 | 118 | honeydew | 43 |
| darkred | 263 | navajowhite2 | 115 | coral | 42 |
| royalblue | 263 | maroon | 111 | darkseagreen3 | 42 |
| darkgreen | 255 | lightpink4 | 109 | antiquewhite2 | 40 |
| darkturquoise | 247 | lavenderblush3 | 108 | coral3 | 40 |
| darkgrey | 243 | darkseagreen4 | 107 | mediumpurple4 | 38 |
| orange | 231 | honeydew1 | 107 | skyblue4 | 37 |
| darkorange | 230 | coral1 | 101 | yellow3 | 37 |
| white | 219 | antiquewhite4 | 100 | sienna4 | 31 |
| skyblue | 209 | coral2 | 99 |  |  |
| saddlebrown | 208 | mediumorchid | 97 |  |  |
| steelblue | 206 | skyblue2 | 95 |  |  |
| paleturquoise | 201 | yellow4 | 94 |  |  |
| violet | 201 | skyblue1 | 89 |  |  |
| darkolivegreen | 187 | lightsteelblue | 86 |  |  |
| darkmagenta | 184 | mediumpurple2 | 86 |  |  |
| **Analysis: SHvsP** |  |  |  |  |  |
| turquoise | 4199 | ivory | 97 | lightpink3 | 55 |
| blue | 2489 | floralwhite | 94 | lavenderblush2 | 54 |
| brown | 1830 | darkorange2 | 93 | darkseagreen3 | 53 |
| yellow | 1282 | bisque4 | 91 | honeydew | 53 |
| green | 1170 | brown4 | 91 | antiquewhite2 | 52 |
| red | 593 | darkslateblue | 90 | coral | 52 |
| black | 537 | plum2 | 90 | coral3 | 52 |
| pink | 469 | thistle2 | 89 | mediumpurple4 | 50 |
| magenta | 424 | salmon4 | 85 | skyblue4 | 49 |
| purple | 391 | thistle1 | 85 | yellow3 | 48 |
| greenyellow | 309 | lightpink4 | 82 | sienna4 | 47 |
| tan | 262 | maroon | 82 | mediumpurple1 | 46 |
| salmon | 247 | navajowhite2 | 82 | orangered1 | 46 |
| cyan | 246 | palevioletred3 | 82 | pink4 | 46 |
| midnightblue | 233 | lavenderblush3 | 81 | indianred3 | 45 |
| lightcyan | 213 | honeydew1 | 78 | lightblue4 | 45 |
| grey60 | 211 | darkseagreen4 | 77 | lightslateblue | 45 |
| lightgreen | 201 | coral1 | 76 | firebrick3 | 41 |
| lightyellow | 197 | antiquewhite4 | 75 | darkolivegreen2 | 40 |
| royalblue | 182 | coral2 | 75 | blue4 | 38 |
| darkred | 176 | mediumorchid | 74 | blueviolet | 38 |
| darkgreen | 174 | skyblue2 | 73 | deeppink | 38 |
| darkturquoise | 144 | skyblue1 | 71 | plum4 | 32 |
| darkgrey | 142 | yellow4 | 71 |  |  |
| orange | 140 | plum | 70 |  |  |
| darkorange | 139 | orangered3 | 68 |  |  |
| white | 137 | lightsteelblue | 66 |  |  |
| skyblue | 136 | mediumpurple2 | 66 |  |  |
| saddlebrown | 135 | lightcoral | 65 |  |  |
| steelblue | 127 | indianred4 | 64 |  |  |
| paleturquoise | 122 | darkolivegreen4 | 63 |  |  |
| violet | 119 | firebrick4 | 63 |  |  |
| darkolivegreen | 114 | blue2 | 62 |  |  |
| darkmagenta | 111 | brown2 | 62 |  |  |
| sienna3 | 107 | darkviolet | 62 |  |  |
| yellowgreen | 107 | plum3 | 61 |  |  |
| skyblue3 | 104 | thistle3 | 61 |  |  |
| orangered4 | 102 | thistle | 59 |  |  |
| plum1 | 102 | salmon2 | 58 |  |  |
| lightcyan1 | 98 | magenta4 | 57 |  |  |
| lightsteelblue1 | 98 | navajowhite1 | 57 |  |  |
| mediumpurple3 | 98 | palevioletred2 | 57 |  |  |
